# Analysis of player heart rate and stroke success in tennis drill scenarios

**DOI:** 10.3389/fphys.2026.1820693

**Published:** 2026-04-30

**Authors:** Ibrahim Cem Balci, Buse Cicek, Irem Sayin, Serkan Salturk, Onur Sarialioglu, Deniz Ozel, Nika Kakulia, Ali Anil Demircali, Huseyin Uvet

**Affiliations:** 1 Department of Mechatronics Engineering, Yildiz Technical University, Istanbul, Türkiye; 2 Department of Mathematics and Science Education, Yildiz Technical University, Istanbul, Türkiye; 3 Department of Informatics, Yildiz Technical University, Istanbul, Türkiye; 4 Department of Biostatistics and Medical Informatics, Akdeniz University Medical School, Antalya, Türkiye; 5 Megasaray Tennis Academy, Antalya, Türkiye; 6 Department of Metabolism, Digestion, and Reproduction, Faculty of Medicine, Imperial College London, London, United Kingdom; 7 Istinye University, Artificial Intelligence Research and Application Center (YZAUM), Istanbul, Türkiye

**Keywords:** athlete performance, heart rate, shot success, sport, statistical analysis, tennis

## Abstract

Heart rate (HR) is a practical indicator of physiological load and arousal, yet its shot by shot relationship with tennis performance remains insufficiently characterized. We quantified associations between HR and stroke outcome and spatial accuracy during a standardized target based drill. The dataset comprised 8,197 shots from 23 players across 93 training sessions. HR was sampled at 1 Hz and temporally aligned to each shot using a fixed ±5 s window around the shot reference time, enabling the extraction of absolute HR levels, short term HR changes and HR intensity zones. Shot outcomes (successful/unsuccessful) and distance to the nearest target area corner were obtained using a camera based tracking system. Successful strokes were generally associated with lower HR levels (*p<* 0.001; small effects, *r* ≈ 0.17). Short term HR dynamics provided limited additional discrimination, with only pre-shot change showing a small difference between outcomes. HR intensity zones were associated with success (*p<* 0.001), with a greater proportion of successful strokes occurring in lower zones (Zones 1-3). Accounting for repeated shots within sessions and players, mixed-effects models were additionally fitted. Higher HR remained associated with lower odds of shot success (OR per 10 bpm = 0.957, 95% CI 0.953-0.961) and a model using heart rate at the shot instant yielded very similar estimates. The continuous distance outcome showed weaker evidence after hierarchical adjustment. Overall, shot-aligned HR monitoring may provide useful contextual information for interpreting performance during precision-focused drills. However, the modest effect sizes suggest that HR should be considered alongside technical and task-related indicators rather than as a primary basis for training decisions.

## 1 Introduction

Tennis is a globally recognized and widely played racquet sport that engages millions of individuals across diverse demographics. Known for its fast paced nature, it requires a high level of skill, endurance and precise coordination. In 2021, the International Tennis Federation estimated 87 million active tennis players across 41 surveyed nations (1.71% of their population) (ITF, 2021). Given this popularity, understanding the key factors that drive successful performance is of great importance to both researchers and coaches.

Success in tennis relies on an interaction of technical, tactical, physical and psychological components. Beyond stroke mechanics and footwork, players must repeatedly execute precision demanding actions under fluctuating physiological load in an intermittent, high intensity sport context (Kovacs, 2006; Fernandez-Fernandez et al., 2009). Accordingly, regulating physiological parameters such as heart rate (HR) and stress responses can meaningfully influence performance quality, especially when fatigue and arousal accumulate across rallies and drills (Lambrich and Muehlbauer, 2022; Kovacs, 2006). Differences in movement speed, agility, strength power qualities and the ability to manage physiological stress and fatigue can therefore lead to measurable differences in competitive outcomes and stroke effectiveness (Ulbricht et al., 2016; Mecheri et al., 2019; Sánchez-Pay et al., 2021).

While individual points in tennis can be decided rapidly due to the sport’s fast paced nature, match outcomes unfold over longer durations, increasing the importance of sustaining technical precision under repeated physical and physiological stress. Physical condition is therefore a key determinant of performance and stroke quality in tennis (Lambrich and Muehlbauer, 2022; Kovacs, 2006; Fernandez-Fernandez et al., 2009). Prior work has linked strength and power characteristics to stroke related and serve related outcomes, including lower limb contributions during serving and serve velocity or stroke performance indicators (Girard et al., 2005; Fett et al., 2020; Lambrich and Muehlbauer, 2022). Speed and acceleration capacities are also critical for court coverage and have been associated with performance related profiles in elite junior players (Ulbricht et al., 2016; Kramer et al., 2021). Agility related factors, including movement preparation and split step mechanics, have likewise been connected to high level performance characteristics (Mecheri et al., 2019). In parallel, endurance and tennis specific on court endurance capacities support the maintenance of performance across prolonged play and intense drill demands (Baiget et al., 2014; Brechbuhl et al., 2018; Kaya and Karahan, 2019; Fernandez-Fernandez et al., 2009). These findings collectively support the view that tennis performance is shaped by multiple interacting fitness dimensions rather than a single physiological component (Sánchez-Pay et al., 2021; Elliott et al., 1990).

Among the physiological variables, HR is an important and practical indicator that can provide broad information about an athlete’s internal physiological load (Kovacs, 2006). Monitoring HR allows a general assessment of exercise intensity and cardiovascular strain during performance (Halson, 2014). It may also provide contextual information about fatigue and arousal related changes during performance, but it should be interpreted as a general physiological marker rather than as a direct standalone measure of specific psychological constructs such as focus (Brouwer et al., 2018).

HR has been widely used in tennis research as a practical proxy of physiological load, particularly in studies examining fatigue related changes in stroke performance (Kovacs, 2006; Reid et al., 2008). Fatigue quantified using HR alongside complementary physiological measures such as blood lactate and electromyography has been associated with reductions in hitting success (Rota et al., 2014), while other work has examined fatigue effects using HR alone, reporting stroke type dependent declines in success rates (Davey et al., 2002) and comparing HR related performance changes between elite and amateur players (Lyons et al., 2011). Most existing studies use post-exercise or session averaged HR summaries rather than aligning HR to individual shots and they rarely incorporate objective camera based spatial accuracy measures. Beyond fatigue, HR has also been used to characterise the physiological demands of training drills: drill intensity has been quantified using HR across different drill types (Reid et al., 2008) and examined alongside measures such as oxygen uptake with HR treated as a key indicator of load (Morais and Bragada, 2022; Bekraoui et al., 2012). While this second line of work evaluates drill demands and, in some cases, broad performance outcomes, it does not capture the instantaneous physiological state in the seconds surrounding each stroke, nor relate that shot level state to success and spatial precision within a controlled target based task.

Related work linking heart rate to performance outcomes extends beyond tennis across a range of sports. Heart rate variability features have been used to differentiate successful and unsuccessful athletes (Stepanyan and Lalayan, 2023), heart rate has been examined in relation to three-point shooting success in basketball (Ardigò et al., 2018) and heart rate variability has been analysed alongside movement intensity profiles in badminton (Yiiong et al., 2019). In table tennis, heart rate responses have been characterised with respect to temporal game structure and stroke techniques under simulated competition conditions (Picabea et al., 2023). Consequently with the tennis literature, these studies support HR as a relevant marker of stress, fatigue and exercise intensity, yet they largely focus on accumulated fatigue, session level summaries, or broader activity segments rather than temporally aligning physiological responses to individual actions. As a result, limited evidence is available on whether the momentary HR state in the seconds surrounding a single stroke is associated with the success and spatial precision of that stroke during a standardized target based tennis drill.

This study examined whether shot-level HR is associated with stroke outcome and spatial accuracy during a standardized target-based tennis drill. Specifically, we investigated whether HR level and HR intensity zones were associated with shot success, whether short-term HR changes provided additional information beyond absolute HR level, and whether HR measures were related to spatial error. By analyzing physiological state in direct temporal relation to individual strokes, this study aims to clarify how variations in cardiovascular load relate to precision performance during tennis practice. We hypothesized that lower HR levels and lower HR intensity zones would be associated with higher shot success and lower spatial error.

## 2 Materials and methods

### 2.1 Study population and dataset

This study was approved by the Ethics Committee for Clinical Research of the Faculty of Medicine, Akdeniz University, with decision number KAEK-200, all research was performed in accordance with relevant guidelines. Informed consent was obtained from all participants and/or their legal guardians. The study investigated 23 players from the Megasaray Tennis Academy, each with a minimum of five years of tennis experience. The sample comprised 16 male and 7 female players, 22 were right handed and 1 was left handed. The participants had an average age of 17.70 ± 4.13 (Mean ± SD) years, weight of 65.18 ± 12.05 (Mean ± SD) kg, height of 174.78 ± 8.19 (Mean ± SD) cm and tennis experience of 10.57 ± 4.95 (Mean ± SD) years. Data were collected across 93 dedicated experimental sessions conducted under standardized conditions, totaling 855 minutes of recorded data. Each session corresponded to a single evaluation block of approximately 10 minutes.

### 2.2 Experimental setup

**Figure 1:**
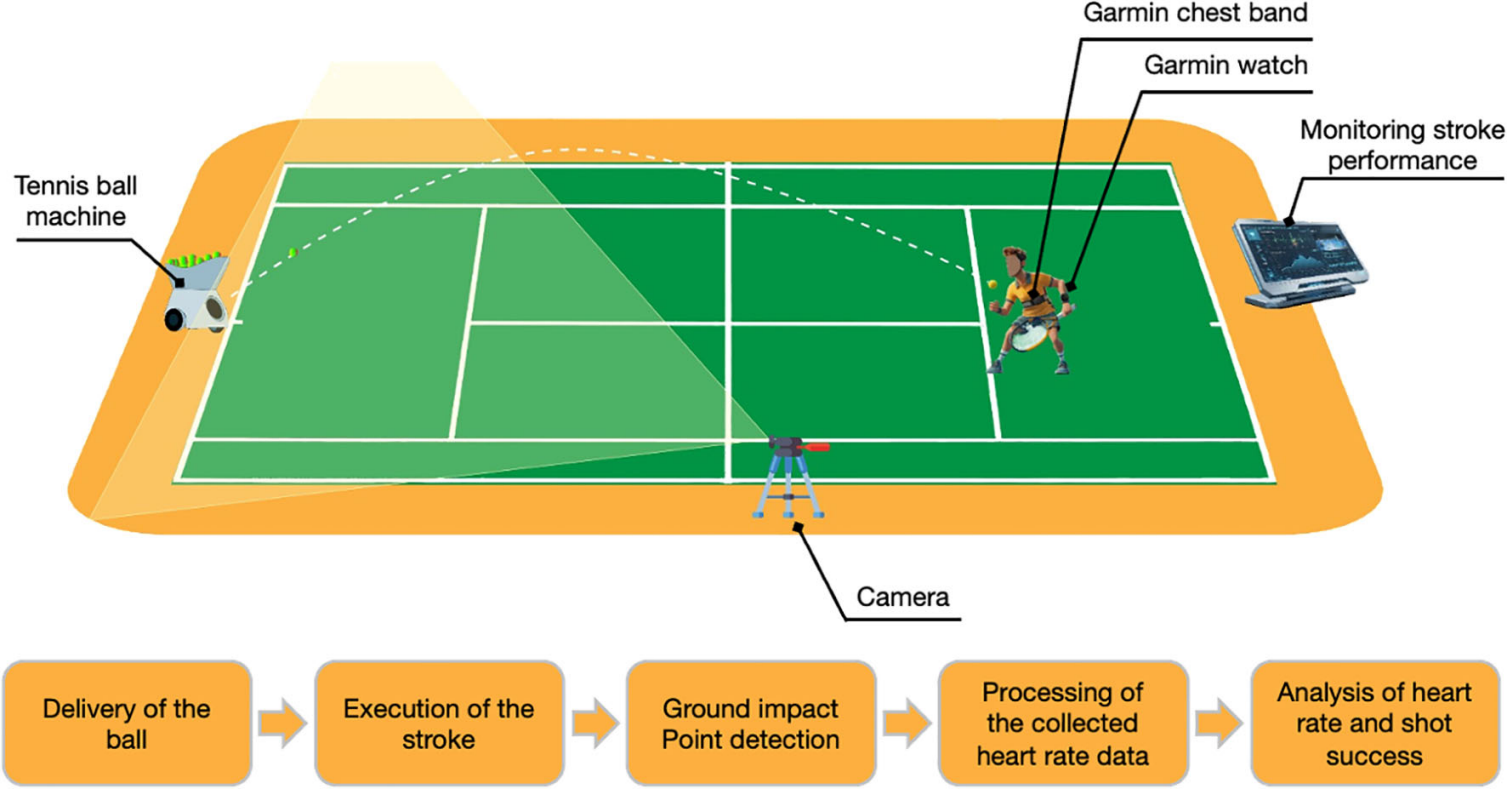
Experimental setup and data collection process.

Data on the success of drill shots and the heart rates of tennis players were collected from 23 players over 93 sessions, yielding a total of 8,197. The data collection process, including the hit scenario and structure, was designed with the assistance of coaches. All sessions were conducted on the outdoor clay court during dry summer conditions. Four target areas measuring 130 × 130 cm were predefined on the opposite court, comprising two deep corner targets positioned near the baseline singles sideline intersections and two short angle forecourt targets positioned nearer the net, close to the service line singles sideline intersections. The target area was selected *a priori* in consultation with academy coaches to create a standardized corner placement task. Rather than reflecting a single universal target standard, its size was chosen to remain within the range reported in established target based tennis assessments and to provide a challenging but attainable precision task for experienced players (Kolman et al., 2017; Furuya et al., 2025). In the forehand drill and backhand drill scenarios, players started from lateral baseline positions and directed the ball to the corresponding deep cross court corner target. In the forehand volleyand backhand volley scenarios, players started from the same forecourt position between the service line and the net and directed the ball to the corresponding short angle forecourt target. In the smash scenarios, players started from a more central forecourt position near the middle of the net and directed the ball to the corresponding short angle forecourt target according to the feed side. In the serve scenario, players served from a fixed service position toward the corresponding short angle forecourt target. Players were instructed to remain close to the predefined starting location, to use only minimal adjustment movement and to land the ball inside the assigned target area as close as possible to the corner of that area nearest the adjacent court line intersection. This nearest corner criterion was used because, in practical tennis terms, balls landing closer to the corners are generally more difficult for an opponent to retrieve than balls landing nearer the center of the same target area. Here, minimizing movement refers to limiting extensive court coverage within each predefined scenario rather than making drill, volley, smash and serve scenarios identical in terms of movement requirements. To standardize the shots, a Staneg Pro 3 ball machine was used with a fixed no spin setting and a feed interval of one ball every 5 seconds within each scenario, feed speed and trajectory were kept constant relative to the predefined player position. **Figure 1** provides a general illustration of the recording setup.

### 2.3 Data collection and instruments

During the data collection phase, heart rate data were recorded using the Garmin Vivoactive 4 Watch and the Garmin Heart Rate Monitor Dual™. The watch, worn on the dominant wrist of each player and the heart rate monitor, positioned under the players’ chests, were synchronized at the start of each session. For the analyses, the HR signal obtained from the chest strap was used as the analytical HR source. Before each session, the computer clock used for video processing and the Garminwatch clock were synchronized. Due to system limitations, heart rate data were collected at a rate of one HR value per second (1 Hz). Given the 1 Hz sampling rate and the known physiological delay of cardiac responses relative to motor actions, these HR variables were interpreted as shot aligned contextual measures of physiological load in the seconds surrounding the shot rather than as instantaneous physiological responses exactly at impact. Recordings were obtained during dedicated experimental sessions rather than during routine training. In each session, players completed one standardized evaluation block of approximately 10 minutes while heart rate and shot outcome data were recorded.

Shot outcomes were classified as unsuccessful when the ball landed outside the target area and successful when it landed inside the target area. Landing point coordinates were obtained from a single court side video stream recorded at 60 fps and processed using our previously described ball impact detection pipeline (Sarıalioğlu et al., 2024). In the present study, the landing labels and landing coordinates used for analysis were semi-automatically checked before analysis. Spatial accuracy was defined as the distance between the ball landing point and the nearest corner of the assigned target area, consistent with the corner directed aiming instruction.

The heart rate data collected from the chest belt were synchronized with shot outcome events using timestamps. The shot reference time (*t* = 0) was defined as the instant of racket ball contact, identified manually from the 60 fps video on a frame by frame basis. Contact timestamps were then aligned with the chest strap HR series using nearest sample matching to the closest available 1 Hz HR value (no interpolation was applied). The manual shot label had a nominal temporal precision of one video frame, whereas the practical precision of HR alignment was limited by the 1 s HR sampling interval. For each shot, the HR value at *t* = 0 and the five preceding and five following 1 s samples were extracted, yielding 11 samples over the fixed [−5, +5] s window. Because successive shots could occur less than 10 s apart, these fixed windows were allowed to overlap between consecutive shots.

### 2.4 Data pre-processing and variable definitions

The dataset included categorical variables (sex, HR zone and shot success) and continuous variables (age, tennis experience, distance to the target and HR-derived features). Descriptive statistics are reported in **Tables 1**, **2**. These variables were used as analytical inputs in the subsequent statistical analyses.

**Table 1:** Descriptive statistics of categorical variables.

Variable	Category	%
Sex	Male	69.6
	Female	30.4
HR Zone	Zone 1	33.9
	Zone 2	30.5
	Zone 3	21.6
	Zone 4	12.3
	Zone 5	1.8
Success	Unsuccessful	75.9
	Successful	24.1

**Table 2:** Descriptive statistics of continuous variables.

Variable	Mean	SD	Min	Max
Age (years)	17.70	4.13	13	32
Experience (years)	10.57	4.95	5	28
Distance (cm)	238.43	145.26	3.86	998.40
*BPM*_−5_ (bpm)	133.98	22.62	78	189
*BPM*_0_ (bpm)	134.28	22.52	78	189
*BPM*_+5_ (bpm)	134.69	22.52	80	189
*BPM*_mean_ (bpm)	134.33	22.50	81.27	188.91
*BPM*_sd_ (bpm)	1.12	0.80	0	11.65
Δ*BPM*_−5→0_ (bpm)	0.28	2.20	-9	17
Δ*BPM*_−5→+5_ (bpm)	0.70	3.29	-13	30
Δ*BPM*_0→+5_ (bpm)	0.43	2.12	-8	18

All predefined scenario types were retained in a common analytic dataset because they shared a common target based precision framework, the same shot level outcome definitions and the same HR alignment procedure. Direct comparisons between scenarios were not the objective of this study. Instead, the analysis examined the overall association between HR and performance across drill situations while adjusting for differences between scenarios.

In addition, shots were performed under multiple standardized drill scenarios; the most frequent scenario types were Forehand Volley (18.5%), Backhand Volley (17.0%) and Forehand Drill (16.3%). The complete distribution of the remaining scenario types is as follows: Forehand Region Smash (15.7%), Backhand Drill (15.9%), Forehand Region Flat Serve (9.0%), Backhand Region Smash (6.8%) and Backhand Region Flat Serve (0.8%).

**Table 1** summarizes the categorical variables used in the study. Among the participants, 69.6% were male and 30.4% were female. Participants were mostly concentrated in HR Zone 1 (33.9%) and HR Zone 2 (30.5%), while the least represented zone was HR Zone 5 (1.8%). According to the success variable, 75.9% of all trials were classified as unsuccessful and 24.1% as successful.

**Table 2** displays the continuous variables and their descriptive statistics. The participants had a mean age of 17.70 years (SD = 4.13) and an average of 10.57 years (SD = 4.95) of playing experience. The mean distance between the ball’s landing point and the nearest target area corner was 238.43 cm (SD = 145.26), indicating substantial variability across trials.

Heart rate measures were extracted relative to the shot reference time (*t* = 0). Specifically, *BPM*_−5_ denotes the HR value 5 seconds before the shot, *BPM*_0_ denotes the HR value at the shot instant and *BPM*_+5_ denotes the HR value 5 seconds after the shot. To summarize the overall HR level and variability around the shot, *BPM*_mean_ and *BPM*_sd_ were computed over the fixed [−5, + 5] second window (i.e., using 11 samples at 1 Hz sampling). Heart rate dynamics were quantified using difference metrics defined as Δ*BPM*_−5→0_ = *BPM*_0_ − *BPM*_−5_, Δ*BPM*_0→+5_ = *BPM*_+5_ − *BPM*_0_ and Δ*BPM*_−5→+5_ = *BPM*_+5_ − *BPM*_−5_.

Normality of each continuous variable was assessed with the one sample Kolmogorov-Smirnov statistic (Kolmogorov, 1933; Smirnov, 1948), which is defined in Equation 1 In this equation, $F_{n}\left(x\right)$ denotes the empirical cumulative distribution function (ECDF) computed from the sample, $F_{0}\left(x\right)$ denotes the reference normal cumulative distribution function (CDF) and sup*_x_*indicates the supremum (i.e., the maximum) over all values of *x*.

(1) $$D=\underset{x}{\sup}\left|F_{n}\left(x\right)-F_{0}\left(x\right)\right|.$$

Because HR-related variables generally deviated from normality, non-parametric methods were used for unadjusted group comparisons and associations. Between group comparisons of successful versus unsuccessful shots were conducted with the Mann-Whitney *U* test (Mann and Whitney, 1947). The test statistic is expressed in Equation 2. Here, *n*_1_ and *n*_2_ are the sample sizes of the two independent groups, *R*_1_ is the sum of ranks for group 1 after pooling and ranking all observations and *U*_1_ is the *U* statistic for group 1. The statistic *U*_2_ is computed analogously by replacing *R*_1_ with the rank-sum of group 2; the reported test statistic is *U* = min(*U*_1_*,U*_2_).

(2) $$U_{1}=n_{1}n_{2}+\frac{n_{1}\left(n_{1}+1\right)}{2}-R_{1},\;U=\min\left(U_{1},U_{2}\right).$$

SPSS provides a standardized normal deviate *Z* for the Mann-Whitney test; we report *Z* and the effect size *r* defined in Equation 3 (Rosenthal, 1986). In Equation 3, *N* = *n*_1_ + *n*_2_ denotes the total number of observations across both groups and |*Z*| is the absolute value of the standardized test statistic.

(3) $$r=\frac{\left|Z\right|}{\sqrt{N}}.$$

Associations between categorical HR intensity zones (HR Zone 1-5) and success status were evaluated using the chi-square test of independence (Pearson, 1900). Because measured *HR*_max_ values were not available, HR zones were defined using the age-predicted maximal heart rate (*HR*_max_ = 220 − age) (Fox, 1971). Specifically, Zone 1 corresponds to 50-60% of *HR*_max_, Zone 2 to 60-70%, Zone 3 to 70-80%, Zone 4 to 80-90% and Zone 5 to 90-100% of *HR*_max_. The chi-square statistic is given in Equation 4. In this equation, *O_ij_*is the observed frequency in row *i* and column *j* of the contingency table, *E_ij_*is the expected frequency under the null hypothesis of independence and the double summation runs over all *r* rows and *c* columns.

(4) $$\chi^{2}=\sum_{i=1}^{r}\sum_{j=1}^{c}\frac{{\left(O_{ij}-E_{ij}\right)}^{2}}{E_{ij}}.$$

Effect size for this association was quantified using Cramér’s *V* as defined in Equation 5 (Cramér, 1999). In Equation 5, *N* is the total number of observations used in the contingency table and *k* = min(*r,c*) is the smaller of the number of rows and columns.

(5) $$V=\sqrt{\frac{\chi^{2}}{N\;\left(k-1\right)}},\;k=\min\left(r,c\right).$$

Because the dataset comprised repeated shots nested within sessions and players, we additionally fitted hierarchical mixed-effects models for formal inference. In all mixed-effects models, scenario type was included as a fixed effect to adjust for heterogeneity in motor and coordinative demands across scenario types, rather than to treat scenario to scenario differences as the primary object of inference. Shot success was analysed using three logistic mixed-effects models with random intercepts for player and session. Model 1 included *BPM*_mean_ together with scenario type, sex and tennis experience as fixed effects. Model 2 replaced *BPM*_mean_ with a within player centered component and a player mean component and additionally included elapsed session time, scenario type, sex and tennis experience as fixed effects. Model 3 replaced *BPM*_mean_ with *BPM*_0_ while retaining scenario type, sex and tennis experience. Continuous distance to target was analysed with a linear mixed-effects model including random intercepts for player and session and scenario as a fixed effect.

## 3 Results

The descriptive shot level statistics are summarized in **Table 1** and **Table 2**. Because the HR variables were non-normally distributed, Mann-Whitney and chi-square analyses are reported below as unadjusted descriptive comparisons. However, because the dataset comprised repeated shots nested within sessions and players, formal inferential interpretation is based on the mixed-effects reanalysis reported after the descriptive results.

Mann-Whitney U test results comparing HR level measures between successful and unsuccessful shots are provided in **Table 3**. Successful shots were associated with lower *BPM*_−5_, *BPM*_0_, *BPM*_+5_ and *BPM*_mean_ values (all *p<* 0.001), with *small* effect sizes (*r* ≈ 0.17). Given the large number of shots, very small effects can yield highly significant p-values. For *BPM*_sd_, the difference was statistically significant (*Z* = −3.21, *p* = 0.001) but trivial in magnitude (*r* = 0.048) and group means were very similar (1.14 vs 1.11 bpm). Because the Mann-Whitney test is rank based, the sign of *Z* depends on rank sum direction in the software output; substantive interpretation should be guided by the group summaries and rank distributions.

**Table 3:** Heart rate level measures by success status (Mann-Whitney U).

Variable	Successful (Mean ± SD)	Unsuccessful (Mean ± SD)	*Z*	*p*	*r*
*BPM*_−5_	129.24 ± 22.08	135.51 ± 22.55	-11.04	<0.001	0.165
*BPM*_0_	129.40 ± 22.12	135.82 ± 22.43	-11.35	<0.001	0.169
*BPM*_+5_	129.82 ± 22.19	136.25 ± 22.38	-11.33	<0.001	0.169
*BPM*_mean_	129.48 ± 22.07	135.88 ± 22.38	-11.33	<0.001	0.169
*BPM*_sd_	1.14 ± 0.76	1.11 ± 0.81	-3.21	0.001	0.048

**Figure 2:**
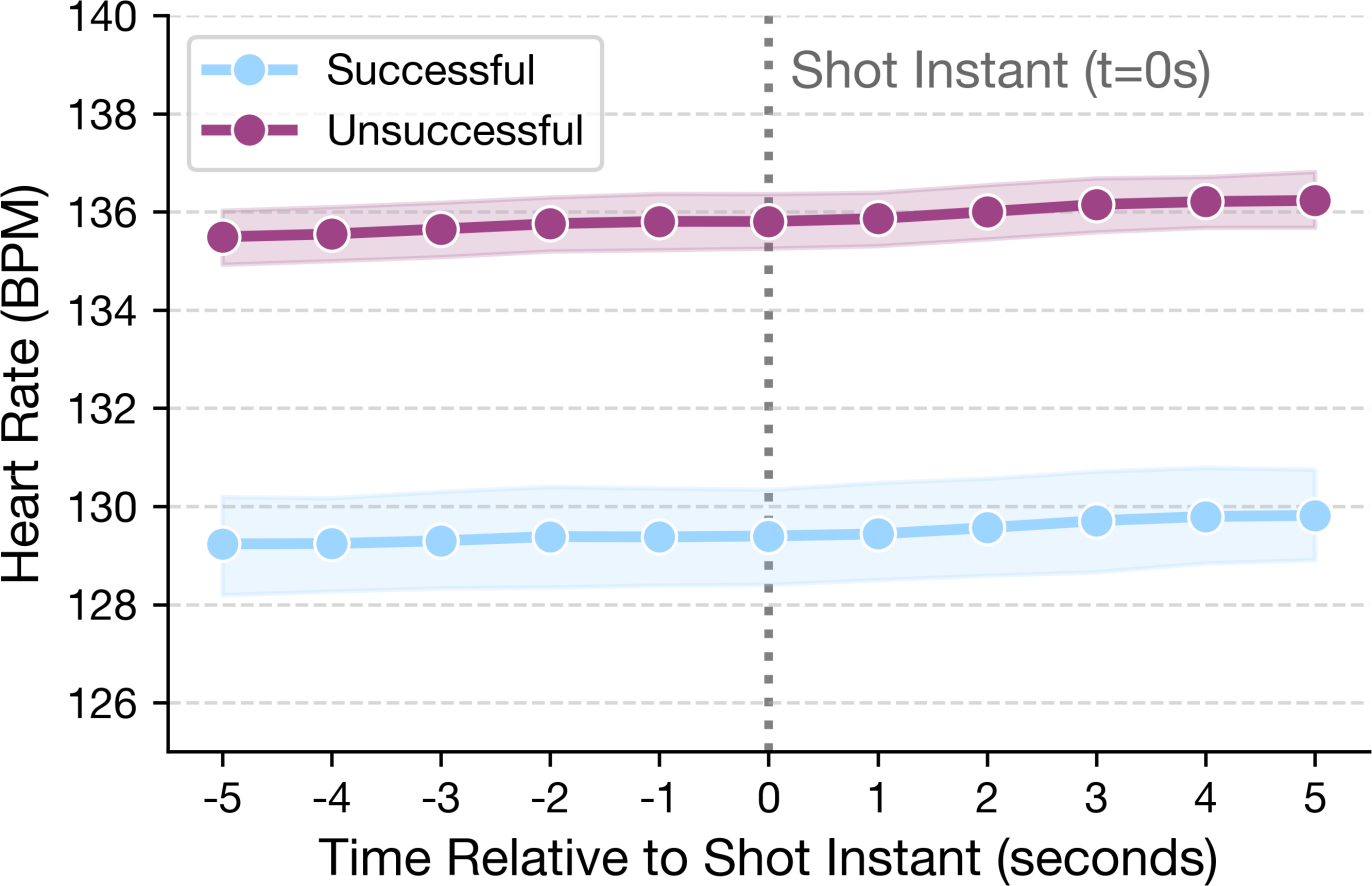
Time series heart rate trajectory from 5s before to 5s after the shot instant.

Group comparisons of the heart rate change metrics are summarized in **Table 4**. The pre to impact change Δ*BPM*_−5→0_ was statistically smaller for successful shots (*p* = 0.024), but the effect size was very small (*r* = 0.033). No statistically significant differences were found for Δ*BPM*_0→+5_ or Δ*BPM*_−5→+5_ (*p >* 0.05). To complement these discrete change metrics, the continuous heart rate trajectory over the entire 11 second window is given in **Figure 2**. Descriptively, mean HR was lower for successful attempts at each plotted time point across the 11 second window. The shaded regions indicate 95% confidence intervals for the two outcome groups.

**Table 4:** Heart rate difference metrics (Δ*BPM*) by success status (Mann-Whitney U).

Variable	Successful (Mean ± SD)	Unsuccessful (Mean ± SD)	*Z*	*p*	*r*
Δ*BPM*_−5→0_	0.16 ± 2.22	0.31 ± 2.19	-2.25	0.024	0.033
Δ*BPM*_0→+5_	0.42 ± 2.14	0.43 ± 2.11	-0.39	0.695	0.006
Δ*BPM*_−5→+5_	0.58 ± 3.20	0.74 ± 3.31	-1.18	0.240	0.017

**Figure 3:**
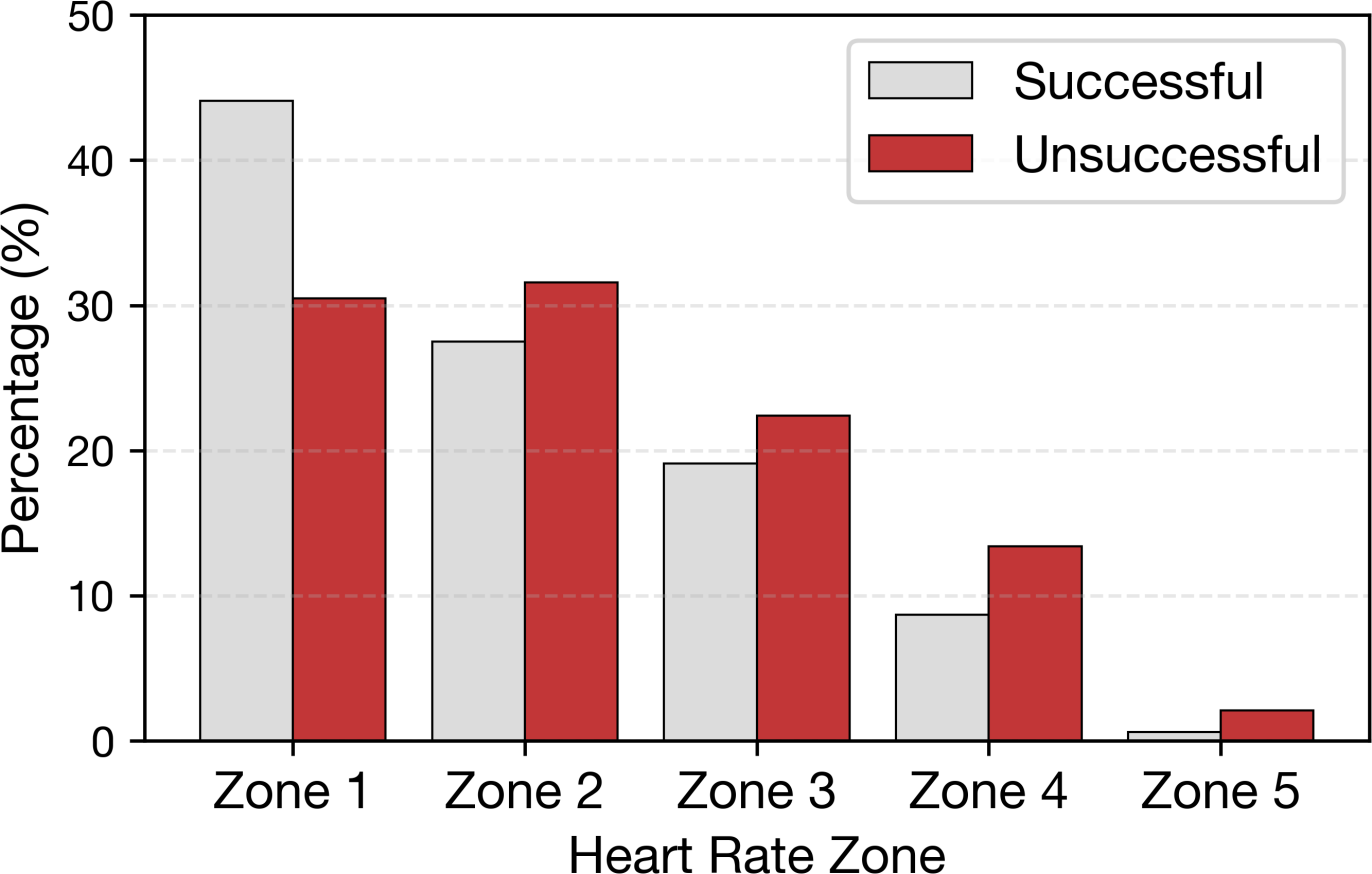
Distribution of success status across HR zones.

**Figure 3** summarizes the distribution of successful and unsuccessful shots across HR zones (within group percentages). The chi-square test indicated a statistically significant association between HR Zone and success (*χ*^2^ = 145.4, *p<* 0.001), with a small effect size (Cramér’s *V* = 0.133). Successful shots were relatively more frequent in lower intensity zones (Zones 1-3), whereas unsuccessful shots accounted for larger shares in higher intensity zones (Zones 4-5). This unadjusted pattern is consistent with lower success proportions at higher HR zones.

To address repeated shots within sessions and players, three logistic mixed-effects models with random intercepts for player and session were additionally fitted for formal inference. In **Table 5**, HR predictors are expressed per 10 bpm and elapsed session time per minute. Model 1 included *BPM*_mean_, scenario, sex and tennis experience. In Model 1, higher *BPM*_mean_ was associated with lower odds of a successful shot (odds ratio (OR) = 0.957, 95% CI 0.953-0.961). Model 2 included a within player deviation term for *BPM*_mean_, a player mean *BPM*_mean_ term, elapsed session time, scenario, sex and tennis experience. In Model 2, both the deviation from the player’s own mean (OR = 0.938, 95% CI 0.911-0.966) and the player’s mean *BPM*_mean_ across analyzed shots (OR = 0.873, 95% CI 0.869-0.876) were associated with lower odds of success, whereas elapsed session time was positively associated with success (OR = 1.028, 95% CI 1.019-1.037), indicating slightly higher odds of success later in the session. Model 3 replaced *BPM*_mean_ with *BPM*_0_ while retaining scenario, sex and tennis experience and yielded very similar estimates (OR = 0.957, 95% CI 0.953-0.961). For the HR predictors in **Table 5**, OR values below 1 indicate lower odds of shot success at higher HR. Approximate z-based *p*-values from the fitted mixed-effects models are also reported in the same table.

**Table 5:** Adjusted odds ratios from mixed-effects logistic models for shot success, accounting for repeated shots within sessions and players.

Model	Predictor	OR (95% CI)	*p*
1	*BPM*_mean_	0.957 (0.953-0.961)	<0.001
2	Deviation of *BPM*_mean_ from the player’s own mean	0.938 (0.911-0.966)	<0.001
2	Player’s mean *BPM*_mean_ across analyzed shots	0.873 (0.869-0.876)	<0.001
2	Elapsed session time	1.028 (1.019-1.037)	<0.001
3	*BPM*_0_	0.957 (0.953-0.961)	<0.001

**Figure 4:**
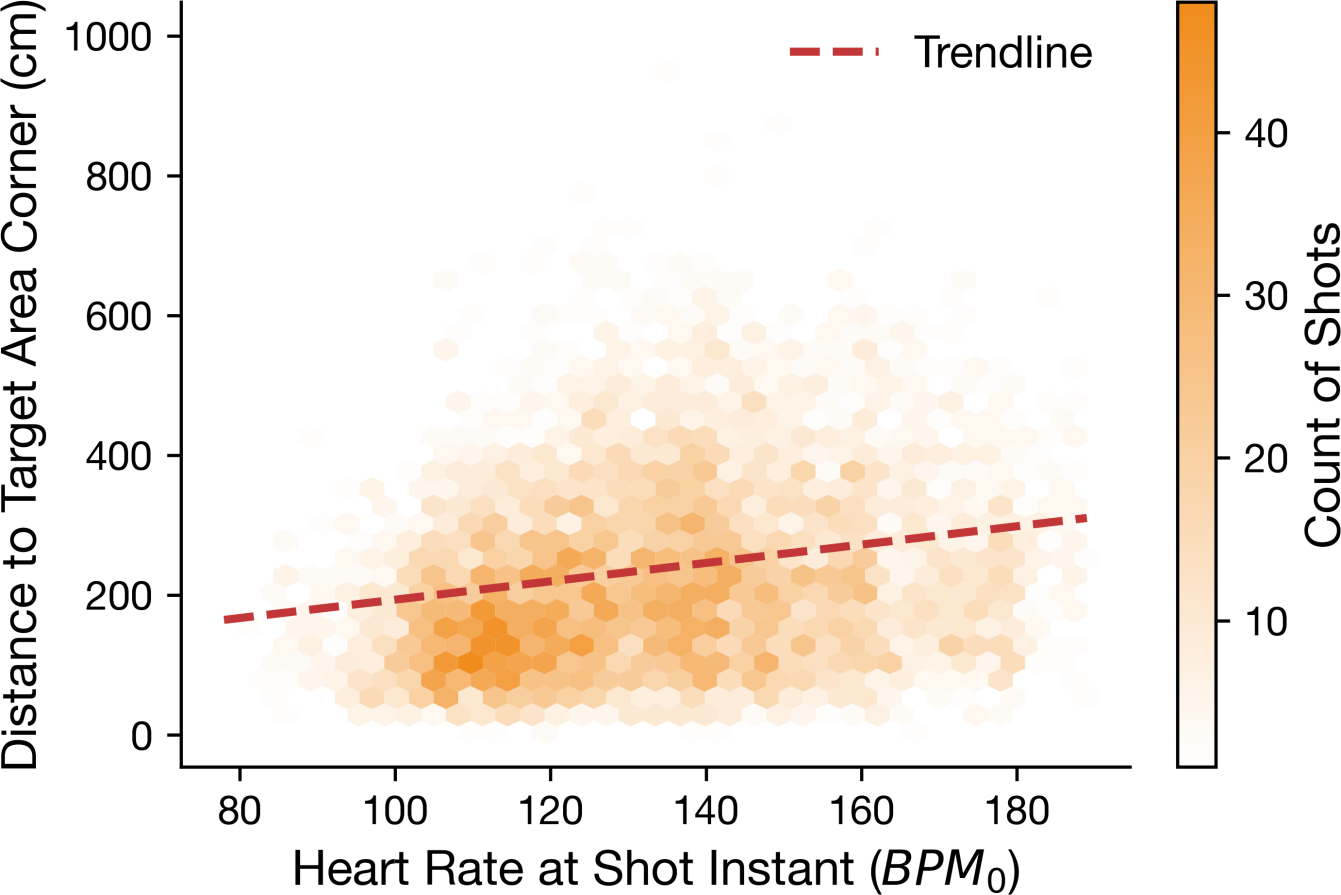
Hexbin density scatter plot for relationship between *BPM*_0_ and distance.

Continuous distance to the nearest target area corner was analysed as a secondary outcome. **Figure 4** provides a descriptive visualization of the bivariate relationship between *BPM*_0_ and distance. In a linear mixed-effects model including random intercepts for player and session and scenario as a fixed effect, *BPM*_mean_ was not clearly associated with distance after hierarchical adjustment (*β* = −1.29 cm per 10 bpm, 95% CI −4.38 to 1.80, *p* = 0.414).

## 4 Discussion

The study examined whether heart rate level, short term HR dynamics around the shot and categorical HR intensity zones were associated with tennis shot success in a standardized target based drill, while also considering the continuous distance metricas a secondary outcome. In the descriptive shot level analyses, successful attempts were generally performed at lower HR levels across the entire 11 second window and they were more frequently observed in lower HR zones. The time series visualization showed a similar descriptive pattern, with lower mean HR values for successful shots at each plotted time point relative to unsuccessful attempts. These descriptive findings provided an initial indication that lower physiological load around the shot may be associated with better performance in this drill setting.

More importantly, the mixed-effects reanalysis showed that this pattern remained after accounting for repeated shots nested within sessions and players. In Model 1, higher *BPM*_mean_ remained associated with lower odds of a successful shot after adjustment for scenario, sex and tennis experience. In Model 2, both temporary increases in HR within players and higher average HR across players were associated with lower odds of success, suggesting that the observed relationship is not explainedsolely by stable differences between players in average HR at the shot level. Model 3, which used *BPM*_0_ instead of *BPM*_mean_, yielded very similar estimates, further supporting the robustness of the main association.

Although the direction of the findings was consistent across several analyses, the magnitude of the effects was modest. Accordingly, HR should be interpreted as one contextual contributor to shot performance rather than as a dominant determinant of outcome in this drill. Tennis performance is inherently multifactorial and technical execution, movement demands, tactical context and player specific characteristics are also likely to influence whether an individual shot is successful. Within that broadercontext, the present findings are broadly consistent with earlier tennis studies showing that increased physiological load or fatigue can accompany reductions in stroke performance across training or match related tasks (Davey et al., 2002; Rota et al., 2014; Lyons et al., 2011).

Absolute HR level appeared to be more informative than short-term HR fluctuations in this dataset. In descriptive comparisons, successful shots were associated with lower *BPM*_−5_, *BPM*_0_, *BPM*_+5_ and *BPM*_mean_ values. In contrast, HR change metrics showed only minimal separation between successful and unsuccessful shots, and *BPM*_sd_ differed only trivially between groups. These patterns suggest that the overall physiological state surrounding the shot may be more informative than brief HR accelerations or decelerations measured within the same short time window. Consistent with this interpretation, the HR zone analysis showed that successful shots occurred more frequently in lower zones and less frequently in higher zones. This descriptive pattern aligns with reduced shot success at higher physiological intensity, although the zone-based results should be interpreted cautiously.

The continuous distance outcome yielded weaker and more variable evidence. Retaining a continuous spatial metric remains methodologically valuable because it captures gradations of performance that a binary success variable can miss. In the present dataset, however, the inferential signal for distance was weaker than for binary shot success. Although the descriptive visualization suggested a positive bivariate relation between *BPM*_0_ and distance, the mixed-effects model for distance was not statistically conclusive after accounting for repeated shots within sessions and players. The most robust inferential signal in the current study therefore concerns binary shot success rather than continuous spatial error.

From an applied perspective, the results suggest that HR monitoring may serve as a useful practical indicator of physiological state during precision drills. Coaches may consider HR alongside technical and task related factors when interpreting fluctuations in shot success, particularly in drills designed to challenge precision under physiological load. At the same time, the modest size of the association suggests that HR should complement, rather than replace, broader performance assessment. In practice, HR monitoring may be most useful for identifying drill contexts in which elevated physiological load coincides with somewhat lower shot success on average, while recognizing that HR is only one component of performance regulation.

Several limitations should be considered. First, HR was sampled at 1 Hz, which may not fully capture rapid beat to beat cardiac fluctuations around the shot. Accordingly, the derived HR variables should be interpreted as shot aligned contextual measures of physiological load in the seconds surrounding the shot rather than as instantaneous physiological responses exactly at racket ball contact. Second, HR intensity zones were defined using age predicted rather than measured *HR*_max_, which is a pragmatic but approximate approach and may have introduced some individual misclassification, particularly in a sample spanning adolescence to adulthood. Third, success was defined using a fixed target area in a controlled drill, future studies could employ variable targets and match like decision constraints.

## 5 Conclusion

This study examined shot by shot associations between heart rate and tennis stroke success and accuracy during a standardized target based drill, with shot success as the primary outcome and continuous distance as a secondary outcome. In the descriptive analyses, successful attempts were generally associated with lower HR levels across the 11 second window surrounding impact and successful shots were relatively more frequent in lower HR zones. Short term HR change metrics and HR variability provided comparatively limited additional separation between successful and unsuccessful shots.

When the repeated measures structure of the dataset was taken into account, the main association remained. In mixed-effects models accounting for repeated shots within sessions and players, higher *BPM*_mean_ was associated with lower odds of shot success after adjustment for scenario, sex and tennis experience. A second model further indicated that both temporary increases in HR within players and higher average HR across players contributed to this association. Model 3, which used *BPM*_0_ instead of *BPM*_mean_, yielded very similar estimates. These findings suggest that the observed relationship is unlikely to be explained solely by pooling all shots into a single dataset.

By contrast, the continuous distance outcome provided weaker evidence after hierarchical adjustment. Although descriptive comparisons indicated a tendency for spatial error to increase with higher HR, the mixed-effects model for distance did not produce statistically conclusive results. Consequently, the most robust inferential finding of the present study relates to binary shot success rather than continuous spatial accuracy. In this drill context, HR may therefore serve as a contextual physiologicalindicator accompanying performance, although its association with outcomes was modest. HR should not be interpreted as a deterministic or standalone predictor of shot success. Instead, HR monitoring may help coaches interpret how physiological load coincides with shot outcomes during precision-focused drills, and should be considered alongside technical, tactical, and task-specific evaluation rather than used in isolation.

## Data Availability

The raw data supporting the conclusions of this article will be made available by the authors, without undue reservation.
